# Evolving Paradigms in Melanoma Therapy

**DOI:** 10.6004/jadpro.2016.7.3.8

**Published:** 2016-04-01

**Authors:** Anthony J. Olszanski, Brianna W. Hoffner

**Affiliations:** Fox Chase Cancer Center, Philadelphia, Pennsylvania, and The Angeles Clinic and Research Institute, Los Angeles, California

The evolution in the treatment of melanoma has been remarkable, moving from surgery, radiotherapy, and chemotherapy to contemporary approaches, which, for the first time, have significantly improved the survival of patients with metastatic disease. At JADPRO Live at APSHO, Anthony J. Olszanski, MD, RPh, of Fox Chase Cancer Center, Philadelphia, Pennsylvania, and Brianna W. Hoffner, MS, ANP-BC, AOCNP®, of The Angeles Clinic and Research Institute, Los Angeles, California, discussed how new agents are used and their associated toxicities managed.

## IMMUNOTHERAPY: FIRST IMPROVEMENT IN OVERALL SURVIVAL

"Perhaps the most important month in the history of metastatic melanoma occurred in October 2015, when three new agents were approved," Dr. Olszanski said. All were immunotherapies, which—given the promise of durable disease control—are changing the treatment paradigm, he said. "We now have patients going out 13 years with no evidence of recurrent disease," he said. "This is amazing in melanoma."

The system via which immunotherapies work involves complex interactions among antigen-presenting cells, T cells, and tumor cells, as well as interplay between positive and negative regulatory signals and between the tumor and stroma. "Immune editing" may allow tumors to be eradicated but they may also evade immune surveillance and grow.

The checkpoint inhibitor ipilimumab (Yervoy) blocks the interaction between the CTLA-4 (cytotoxic T-lymphocyte–associated protein 4) receptor on T cells and B7 on antigen presenting cells, thus keeping T cells stimulated and fighting the tumor. In pivotal trials, ipilimumab improved overall survival, as compared with the older treatments dacarbazine and the gp100 vaccine (Robert et al., 2011; Hodi et al., 2010).

"For the first time ever, a drug improved survival in metastatic melanoma, and the tail of the curve flattened, suggesting that we increased the population of patients achieving durable disease control," he said.

## ANTI–PD-1 AGENTS ENTER THE PICTURE

The two monoclonal antibodies approved by the US Food and Drug Administration (FDA) targeting the programmed cell death protein 1 (PD-1) also work by allowing the T cell to remain effective. The PD-1 inhibitors substantially improve survival and produce durable responses in a subset of patients. Nivolumab (Opdivo) led to a 58% reduction in mortality compared with dacarbazine (Robert et al., 2015a), and pembrolizumab (Keytruda) reduced mortality by 27% over ipilimumab (Robert et al., 2015b).

According to Dr. Olszanski, these outcomes are "truly monumental in the treatment of metastatic melanoma" and are even better than those achieved with ipilimumab. The greatest efficacy is observed, however, when the two forms of immunotherapy are combined.

Nivolumab plus ipilimumab led to a 58% reduction in mortality (Larkin et al., 2015). "These are amazing hazard ratios, suggesting that we are bettering the outcomes for the majority of the population," he noted.

Recently added to the immunotherapy arsenal is the injectable oncolytic virus talimogene laherparepvec (T-VEC; Imlygic), which is approved for patients with cutaneous, subcutaneous, or nodal lesions which are recurrent after surgical resection. Injection of T-VEC may stimulate an immune response that regresses not only the injected lesions but distant lesions as well.

## IMMUNE-RELATED ADVERSE EVENTS

As a result of upregulation of the immune system, patients can experience a variety of adverse events caused by inflammation and off-target effects, said Ms. Hoffner. "We are seeing toxicities we didn’t really know existed before," she added.

Dermatitis is observed in up to 40% of patients receiving ipilimumab and 30% receiving anti–PD-1 agents. With ipilimumab, this rash can be severe—Stevens-Johnson syndrome and toxic epidermis necrolysis have been observed.

Mild-to-moderate dermatitis can be managed symptomatically. Shower time should be brief; patients may use unscented creams/emollients for moisturizing and an antihistamine or 1% hydrocortisone cream for itching. More severe cases may respond to moderate-potency triamcinolone 0.1% cream or moderate-dose parenteral prednisone (or equivalent) at 0.5 mg/kg/day, and they should be tapered gradually.

Diarrhea or colitis is the most common and potentially the most serious complication of ipilimumab, reported by nearly one-third of patients receiving it, with 6% being severe cases. It is less common (20%) with anti–PD-1 agents.

Clinicians should always rule out other causes of diarrhea and also realize that patients may have colitis (inflammation of the colon) without diarrhea. Management of colitis is shown in the Table.

**Table:**
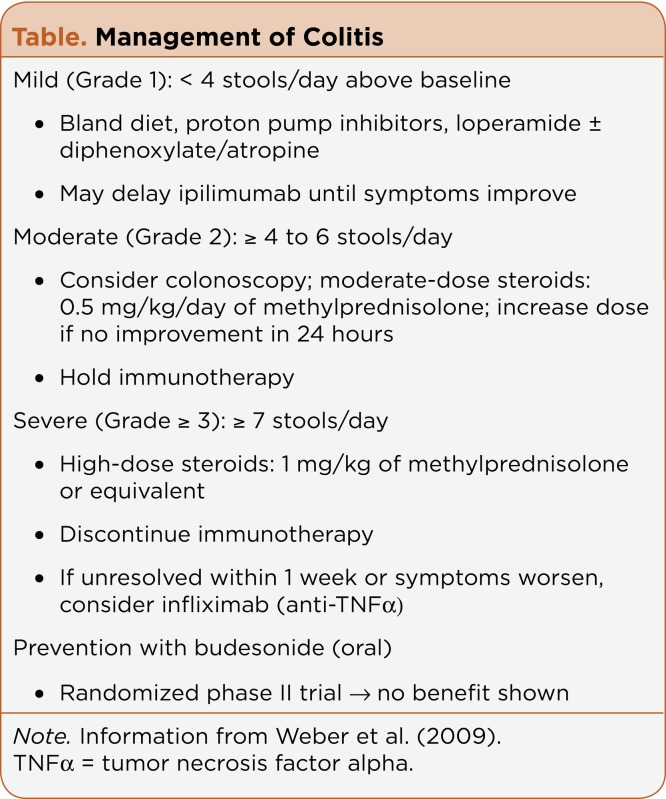
Management of Colitis

Hepatotoxicity is uncommon, but its incidence is increasing as a result of combination immunotherapy and can be life-threatening, Ms. Hoffner pointed out. Symptoms include abdominal bloating or pain, dyspepsia, jaundice, and nausea, or they can be vague or altogether absent. Liver function tests should be performed at baseline and prior to each dose. For hepatotoxicity grade ≥ 3, she recommended corticosteroids and, if necessary, mycophenolate.

Endocrinopathy is the one immune-related adverse event that may not be reversible, but it can be controlled. Hypothyroidism is seen in 8% of patients on anti–PD-1 agents (Merck, 2015). A thyroid-stimulating hormone (TSH) test should be performed every 12 weeks, and Ms. Hoffner recommends follow-up with more specific tests if endocrine symptoms persist. Patients should also be monitored for symptoms associated with pituitary and adrenal disease. Treatment of endocrinopathies requires appropriate hormone replacement, corticosteroids, and possibly drug discontinuation.

## *BRAF* MUTATION: KINASE INHIBITION

Kinase inhibitors targeting the *BRAF* mutation were the second class of drugs to improve OS among the ~50% of patients with that mutation. Vemurafenib (Zelboraf) reduced mortality by 30%, compared with dacarbazine (Chapman et al., 2012). Median overall survival was 13.6 months, vs. 9.7, and response rates were improved almost fivefold.

The second approved BRAF inhibitor, dabrafenib (Tafinlar), demonstrated a 65% reduction in mortality over dacarbazine (Hauschild et al., 2012), Dr. Olszanski reported. However, he cautioned, although approximately 50% of patients respond to treatment, and do so fairly rapidly, *BRAF*-mutated tumors often acquire resistance to BRAF inhibitors within about 6–7 months. By targeting an additional pathway with a MEK inhibitor, response rates reach "an astounding" 75%, he said, and the duration of response almost doubles (Flaherty et al., 2012). The combination regimen of a BRAF inhibitor plus a CTLA-4 inhibitor also improves outcomes, Dr. Olszanski noted. "Perhaps we are increasing both response rates and durability through these new therapies to get closer to the ’C’ word—not ’cancer,’ but ’cure,’ " he commented.

## MANAGING TOXICITIES WITH BRAF/MEK INHIBITORS

Clinicians should be alert to the unique side effects of BRAF and MEK inhibitors. The main toxicity is dermatologic, including photosensitivity and skin cancers. "While some patients experience no dermatologic toxicity, others can get a grade 3 blistering sunburn within 5 minutes," Ms. Hoffner noted.

While on these agents, patients should avoid the sun and use protective clothing and sunscreen on all exposed body parts. Patients should also be monitored every 2 months for the development of squamous cell carcinoma and new melanomas. Other side effects can be arthralgias, nausea, fatigue, uveitis (ocular inflammation), QTc prolongation, hepatotoxicity, and alopecia.

Unique to MEK inhibitors is retinal vein occlusion—which mandates discontinuation of the drug—and retinal pigment epithelial detachment.

Cardiac abnormalities and grade ≥ 3 elevations in liver function test levels warrant dose reduction or discontinuation of the drug until they resolve. Electrocardiograms should be obtained at baseline, day 15, monthly for 3 months, and then every 3 months.

With MEK inhibitors, advanced practitioners should watch for bone marrow suppression, and complete blood cell counts should be taken at every visit. Changes in left ventricular ejection fraction warrant dose reduction or hold and close follow-up. Diarrhea can be significant on MEK inhibitors, and treatment is the same as for ipilimumab.

When BRAF and MEK inhibitors are combined, side effects are actually less severe than with single agents, and response rates and duration of response are longer, she added.

The main thing to watch for with combined BRAF/MEK inhibition is pyrexia, observed in 70% of patients. "Patients will go to the emergency room and get evaluated for neutropenic fever, but this has nothing to do with infection," Ms. Hoffner said. "Discuss this prior to treatment, and tell the patient to call you first."

Dr. Olszanski commented on the management of toxicities. "Advanced practitioners play an important role in managing immune-related and kinase-related adverse events, which can be deva-stating and even life-threatening. We need someone watching these patients like a hawk," he said.

Although recent advances have revolutionized melanoma treatment, he added, "We still have a long way to go to help the majority of patients, and research and clinical trials are the answer. I do think we will get there." 
